# Macrophytes shape trophic niche variation among generalist fishes

**DOI:** 10.1371/journal.pone.0177114

**Published:** 2017-05-09

**Authors:** Ivana Vejříková, Antti P. Eloranta, Lukáš Vejřík, Marek Šmejkal, Martin Čech, Zuzana Sajdlová, Jaroslava Frouzová, Mikko Kiljunen, Jiří Peterka

**Affiliations:** 1 Biology Centre of the Czech Academy of Sciences, Institute of Hydrobiology, Na Sádkách 7, České Budějovice, Czech Republic; 2 Faculty of Science, University of South Bohemia in České Budějovice, Branišovská 31, České Budějovice, Czech Republic; 3 Norwegian Institute for Nature Research, Sluppen, Trondheim Norway; 4 University of Jyväskylä, Department of Biological and Environmental Science, Jyväskylä, Finland; Universitat Trier, GERMANY

## Abstract

Generalist species commonly have a fundamental role in ecosystems as they can integrate spatially distinct habitats and food-web compartments, as well as control the composition, abundance and behavior of organisms at different trophic levels. Generalist populations typically consist of specialized individuals, but the potential for and hence degree of individual niche variation can be largely determined by habitat complexity. We compared individual niche variation within three generalist fishes between two comparable lakes in the Czech Republic differing in macrophyte cover, i.e. macrophyte-rich Milada and macrophyte-poor Most. We tested the hypothesis that large individual niche variation among generalist fishes is facilitated by the presence of macrophytes, which provides niches and predation shelter for fish and their prey items. Based on results from stable nitrogen (δ^15^N) and carbon (δ^13^C) isotopic mixing models, perch (*Perca fluviatilis* L.) and rudd (*Scardinius erythrophthalmus* (L.)) showed larger individual variation (i.e., variance) in trophic position in Milada as compared to Most, whereas no significant between-lake differences were observed for roach (*Rutilus rutilus* (L.)). Contrary to our hypothesis, all the three species showed significantly lower individual variation in the relative reliance on littoral food resources in Milada than in Most. Rudd relied significantly more whereas perch and roach relied less on littoral food resources in Milada than in Most, likely due to prevalent herbivory by rudd and prevalent zooplanktivory by perch and roach in the macrophyte-rich Milada as compared to macrophyte-poor Most. Our study demonstrates how the succession of macrophyte vegetation, via its effects on the physical and biological complexity of the littoral zone and on the availability of small prey fish and zooplankton, can strongly influence individual niche variation among generalist fishes with different ontogenetic trajectories, and hence the overall food-web structures in lake ecosystems.

## Introduction

Generalist species that feed on multiple trophic levels (*cf*. [1]) commonly have a fundamental role in ecosystems. Generalists can, for instance, regulate the abundance, composition and niche use of organisms at lower and higher trophic levels and also integrate spatially distinct habitats and food-web compartments [2], thereby affecting the structure and stability of food webs [3,4]. Generalist populations with wide trophic niche typically consist of highly specialized individuals that only use a subset of the entire population niche [5]. Such individual specialization can, in turn, have complex population-, community- and ecosystem-level effects, including speciation processes, intra- and inter-specific interactions as well as nutrient cycles and energy flow ([6–8] and references therein). However, the occurrence and degree of individual niche variation, and hence potential for speciation and habitat coupling, within generalist populations is commonly controlled by niche availability and ecological opportunities [9–11].

In lakes, the niche availability and ecological opportunities are strongly controlled by the presence and extent of macrophyte vegetation, both through the direct effects on habitat complexity and the overall ecosystem productivity and also indirectly through reduced predatory effects (*e*.*g*. [12,13] and references therein). Besides affecting the abundance, size and community composition of zooplankton, benthic macroinvertebrates and small fish [12,14,15], macrophytes can also directly contribute to the diet of generalist fishes [13,16]. The effects of macrophyte vegetation on individual niche variation among generalist fishes can largely be shaped by the species’ foraging strategy and ontogenetic trajectories. However, there is limited empirical evidence of how macrophyte vegetation affects individual niche variation and hence the overall population-level niche width of generalist fishes. Here, we use a novel opportunity to compare how different succession stages (*i*.*e*., abundance) of macrophyte beds influence trophic niche width of three generalist fishes with contrasting foraging strategies. Perch (*Perca fluviatilis* L.), roach (*Rutilus rutilus* (L.)) and rudd (*Scardinius erythrophthalmus* (L.)) are all generalist and widely spread fishes [17], the former two species commonly dominating the fish communities in European lakes [18–20]. However, the three species show fundamental differences in their foraging strategies. Perch commonly undergo ontogenetic dietary shift from zooplankton to benthic invertebrates and finally to piscivory [21,22], whereas roach and particularly rudd become increasingly herbivorous with increasing size [17,23,24]. Hence, the species can be expected to show markedly different responses to the presence or absence of macrophytes, both in terms of the trophic position occupied by the populations and individuals within, as well as in terms of their use of littoral and pelagic food resources.

The competitive coexistence of perch and roach is one of the most extensively studied examples of intra- and inter-specific interactions among generalist fishes. A general succession from dominance of perch to roach with increasing productivity has been documented for lakes of different trophic states [18,25,26], as well as within reservoirs with pronounced longitudinal gradients [27,28]. Roach are efficient zooplanktivores, but may also feed on macroinvertebrates and non-animal food. Perch are ontogenetic carnivores and start their lives by feeding on zooplankton and later shift to macroinvertebrates to finally become piscivorous [21]. Both species mutually influence each other by way of asymmetrical competitive and predator-prey interactions. Roach are more efficient zooplanktivores in open water and thus force young-of-the-year perch to ingest benthic macroinvertebrates earlier in the season if zooplankton resources are reduced [29,30]. This leads to increased intra-specific competition between perch age classes and thereby to reduced growth and potential to a piscivorous niche shift [30]. In some lakes, perch and roach show marked niche segregation with perch feeding mainly on benthos among macrophyte vegetation and roach feeding on zooplankton in the pelagic areas [29]. However, habitat complexity can fundamentally affect intra- and inter-specific interactions among perch and roach. Structured habitats offer high biomasses of macroinvertebrates [31,32] and thereby facilitate competitive dominance of the more efficient benthivorous perch [29,33]. Moreover, perch is also more efficient zooplanktivore in structured habitats as compared to roach [34]. Consequently, the presence of structurally complex habitats, such as littoral macrophytes, generally reduces the bottlenecks for ontogenetic niche shifts and increases the growth of perch, even if perch and roach are forced to coexist in these sheltered habitats due to the presence of piscivorous predators [32,35]. Although the perch-roach interactions are well studied, there is limited empirical evidence of how macrophyte vegetation can influence trophic niche width of generalist fishes, including also rudd.

We compared isotopic niche widths of generalist perch, roach and rudd between two deep, post-mining lakes in northern Czech Republic: Lake Milada with abundant macrophyte vegetation and Lake Most where macrophytes are practically absent. In Milada, macrophytes evidently increase the structural complexity of the littoral zone and hence provide niches and shelter against fish predation for various invertebrate taxa [13]. This high physical and biological complexity may hence support a more complex food-web structure in Milada as compared to the macrophyte-poor Most. However, the three generalist fishes likely utilize different trophic niches and may also respond differently to between-lake differences in niche availability. Therefore, we raised three main hypotheses: i) Firstly, due to their different foraging strategies and ontogenetic trajectories, we expected significant niche segregation between the three generalist species, with perch occupying the highest trophic position due to piscivory and rudd occupying the lowest trophic position and relying most heavily on littoral food resources due to herbivory. ii) Secondly, we expected that the abundant macrophyte vegetation in Milada would facilitate higher trophic variability within the generalist fish populations than in the macrophyte-poor Most, as individuals can utilize a wider selection of niches (*i*.*e*., prey items and habitats) in the former ecosystem. iii) Thirdly, we expected perch to be particularly responsive to higher niche availability since, unlike more invertivorous and herbivorous roach and rudd, perch can shift to piscivory and utilize small prey fish, which in turn are expected to be more abundant in the macrophyte-rich Milada.

## Materials and methods

### Study lakes

**Fig 1:**
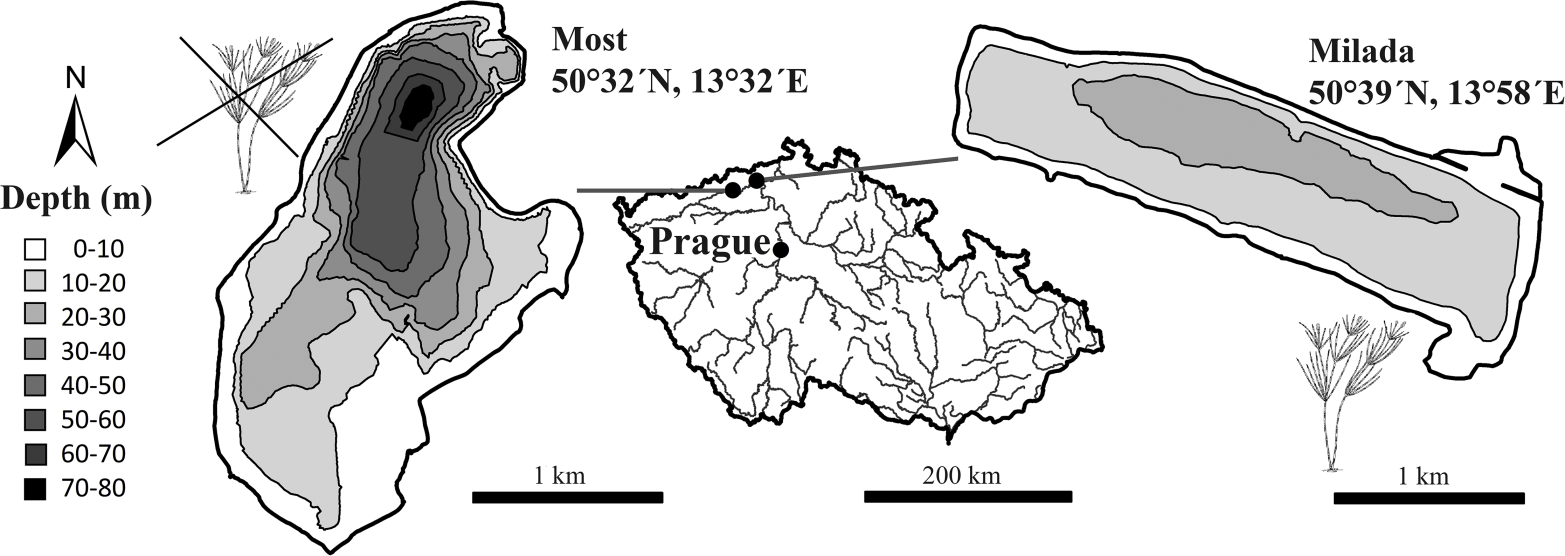
A map showing the location and bathymetric maps of macrophyte-rich Milada Lake and macrophyte-poor Most Lake in northern Czech Republic.

The study was conducted in two newly created comparable post-mining lakes in northern Czech Republic, Milada and Most (Fig 1), with a permission of the owner of the study sites, Palivový kombinát Ústí, státní podnik. The lakes originate from flooding of restored brown coal opencast mines. Aquatic restoration of Milada was performed in years 2001–2011 and nowadays several species of macrophytes and macroalgae are present in high biomasses to a depth of 12 m (average vegetation cover of > 65% at 0–12 m depth, consisting mainly of *Potamogeton* sp., *Myriophyllum* sp. and *Chara* sp.). Restoration of Most was performed in years 2008–2014, but no abundant macrophyte vegetation has been developed (average vegetation cover of < 1% at 0–6 m depth) (see [36] for more details). In years 2013–2014, the three study species composed in abundance 94–96% and 64–65% of the total catches in Milada and Most, respectively. The relative abundances of the species showed no significant between-lake differences (Wilcoxon matched pairs test: *p* = 0.9, Z_6_ = 0.104; S1 Table), with perch and roach being consistently the most abundant and rudd being the least abundant species. Other fishes present in both lakes are ruffe (*Gymnocephalus cernua* (L.)), pike (*Esox lucius* L.), European catfish (*Silurus glanis* L.) and tench (*Tinca tinca* (L.)), whereas pikeperch (*Sander lucioperca* (L.)), asp (*Leuciscus aspius* (L.)) and Prussian carp (*Carassius gibelio* (Bloch)) are only found in Milada and maraena whitefish (*Coregonus maraena* (Bloch)) are only found in Most.

### Fish sampling

Animal treatment was performed in accordance with the guidelines and permission from the Experimental Animal Welfare Commission under the Ministry of Agriculture of the Czech Republic (Ref. No. CZ 01679). The work was approved by the Ethics Committee of the Czech Academy of Sciences. All sampling procedures and experimental manipulations were approved by the Czech Academy of Sciences. The field study did not involve endangered or protected species.

Fish for stable isotope analysis (SIA) were sampled in September 2013 and 2014 by benthic (height 1.5 m, length 30 m) and pelagic (height 3 m, length 30 m) multi-mesh gillnets (12 mesh sizes ranging from 5 to 55 mm knot-to-knot, [37]) in both lakes. The gillnets were set overnight (2 h before sunset and lifted 2 h after sunrise) at depths of 0–3, 3–6, 6–9 and 9–12 m at two benthic and one pelagic localities in three repetitions for both types of gillnets in both lakes and years. Altogether 36 gillnets (24 benthic and 12 pelagic) were set in each lake and year, summing to a total of 144 gillnet nights and 8,640 m^2^ gillnet area. All captured fish were immediately anaesthetized by a lethal dose of tricainemethanesulfonate (MS–222, Sigma Aldrich Co.), identified, measured, weighed and dissected for gut content analysis. Muscle tissues from randomly chosen perch, roach and rudd individuals were stored frozen at –20°C prior to final preparation for SIA.

### Sampling of food sources for SIA

Littoral and pelagic basal resources (macrophytes and macroalgae,particulate organic matter, POM) and invertebrates (benthic macroinvertebrates, zooplankton) were sampled for SIA to study the overall food-web structure in both study lakes and to estimate the trophic position and the relative reliance of generalist fishes on littoral *versus* pelagic food resources using two-source isotopic mixing models (*cf*. [38,39]). Macrophytes, macroalgae, POM and benthic macroinvertebrates were collected in September 2013 and 2014, whereas zooplankton was sampled in September 2013 and in March, May, July, September and November 2014. Macrophytes, macroalgae and benthic macroinvertebrates were collected by two SCUBA divers, using plastic corer in case of macroinvertebrates. Pelagic POM samples were obtained by filtering 20 L of surface water (collected from the uppermost 5 m) through a 50-μm sieve to prevent contamination by animal matter prior to the filtration under pressure through a glass microfiber filter (GF/C 1.2 μm; Whatham, U.S.A.). Zooplankton was sampled with a 100-μm mesh plankton net by several vertical hauls throughout the uppermost 20 m of the water column. All littoral and pelagic food sources were sorted, cleaned of detritus and other unwanted material, identified to genus or family level and stored frozen at –20°C prior to final preparation for SIA. Only soft body tissue was dissected from molluscs and trichopterans with cases.

### Stable isotope analyses

All frozen fish, invertebrate and basal resource SIA samples were dried at 60°C for 48 h and ground into a homogenous powder using a ball-mill Retsch MM 200 (Retsch GmbH, Haan, Germany). Small subsamples (0.520–0.770 mg) were weighed into tin cups for the analysis of δ^15^N and δ^13^C. All SIA were conducted using a FlashEA 1112 elemental analyser coupled to a Finnigan DELTA^plus^ Advantage mass spectrometer (Thermo Fisher Scientific Corporation, Waltham, MA, U.S.A.) at the University of Jyväskylä, Finland. Stable nitrogen and carbon ratios are expressed as delta values (δ^15^N and δ^13^C, respectively) relative to the international standards for nitrogen (atmospheric nitrogen) and carbon (Vienna PeeDeeBelemnite). Analytical precision was always better than 0.20‰ for both isotopes, and was based on standard deviation of repeated analysis of working standards (pike white muscle tissue and birch leaves) inserted in each run after every five samples. As C:N ratios were consistently lower than 3.5 (*i*.*e*., 96% of cases), obtained stable isotope values of fish were not lipid corrected [40].

In this study, SIA was applied to compare individual niche variation within the generalist fishes in macrophyte-rich Milada and macrophyte-poor Most. Stable nitrogen (δ^15^N) and carbon (δ^13^C) isotopes are widely used to study lake food webs and to estimate the long-term diet of consumers [39] as well as niche width of consumer populations [41,42]. While δ^15^N values indicate the trophic position of an organism in the food web, the δ^13^C values reflect the long-term, assimilated carbon sources of consumers, such as the relative proportions of littoral (benthic) *versus* pelagic (planktonic) food for fish [39]. Hence, SIA provides a powerful tool to study food-web complexity (*e*.*g*., food-chain length) and energy flow pathways in the ecosystems.

Primary producers and consumers can show marked differences in δ^15^N and δ^13^C values between different sites [43,44]. Therefore, the δ^15^N and δ^13^C values of higher consumers, such as fish, need to be standardized with the isotopic baselines prior to comparisons across ecosystems. Here, the fish δ^15^N and δ^13^C values were standardized across the study lakes by estimating the trophic position (*TP*) and the relative reliance on littoral *versus* pelagic carbon sources (hereafter littoral reliance, *LR*), respectively, using the two-source isotopic mixing models described in [38]. The mean δ^15^N and δ^13^C values of macrophytes (*i*.*e*. *Myriophyllum* sp., *Potamogeton* sp., *Elodea* sp.) together with macroalgae (*i*.*e*. *Chara* sp.) and of pelagic POM were used as the littoral and pelagic isotopic baselines, respectively. Long-lived primary consumers, such as algae-grazing snails and filtrate feeding zooplankton or clams, are commonly used for estimating isotopic baselines since they often show lower spatial and temporal variation in δ^15^N and δ^13^C as compared to primary produces [38]. However, in the present study, it is more justified to use macrophytes and macroalgae as the littoral baseline since some of the study species (*i*.*e*., roach and particularly rudd) are facultative herbivores and thus can utilize these plant foods with the most elevated δ^13^C values. For the *TP* and *LR* computations, the commonly used trophic fractionation factors of 3.4‰ for δ^15^N and 0.4‰ for δ^13^C were applied [38].

### Statistical analyses

Depending on normality of the data, either parametric *t*-tests or non-parametric Mann-Whitney *U*-tests were used to test between-lake differences in standard length, trophic position (*TP*) and littoral reliance (*LR*) of the generalist fishes. Analysis of variance (ANOVA) with Tukey’s HSD (honest significant difference) pairwise comparisons were used to test differences in *TP* and *LR* between the three species (*i*.*e*., inter-specific niche segregation within the lakes). Between-lake differences in the trophic niche width of the generalist species (measured as variance of *TP* and *LR*) were tested with Levene’s test for equality of variances, which takes into account differences in sample sizes and is robust to non-normality. All statistical analyses were done in R 3.1.1 [45].

## Results

### Interspecific niche segregation

**Fig 2:**
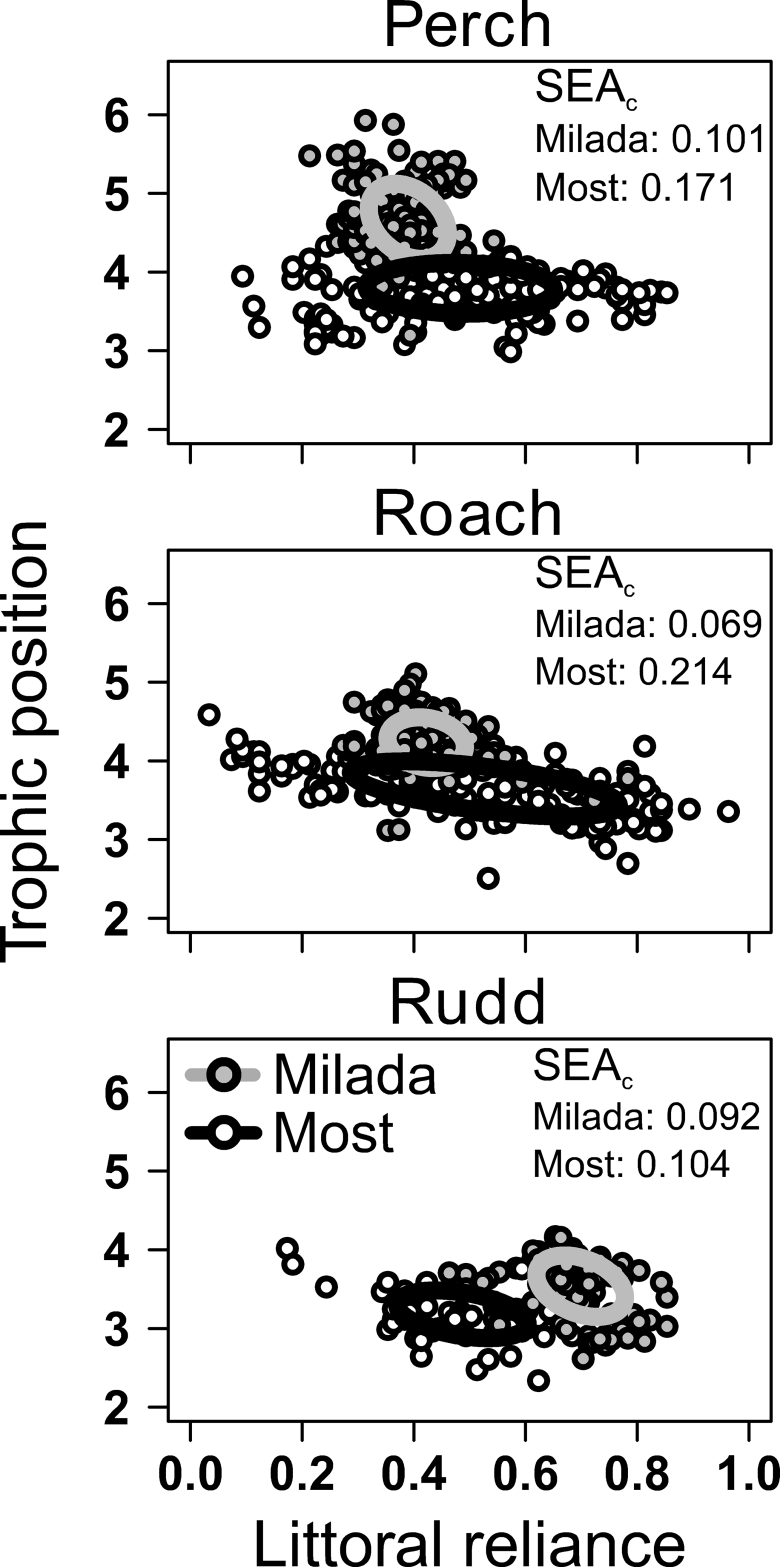
Trophic position versus relative littoral reliance of generalist perch, roach and rudd in the macrophyte-rich Milada (grey dots) and macrophyte-poor Most (white dots). The trophic position (*i*.*e*., the average position relative to primary producers at which an organism feeds) and the littoral reliance (*i*.*e*., the proportion of assimilated food obtained from the littoral habitat) are based on the isotopic values of fish muscle tissue and of the littoral and pelagic basal resources (see Materials and methods and [38] for details). The ellipses depict the core isotopic niches of the fish populations estimated as the sample-size corrected standard ellipse areas (SEAc; estimates shown in the figures; see [68] for details).

The three generalist fishes showed significant niche segregation in terms of their trophic position both in the macrophyte-rich Milada (F_2,423_ = 200.1, *p* < 0.001) and in the macrophyte-poor Most (F_2,336_ = 73.6, *p* < 0.001). In both lakes, perch occupied on average the highest trophic position whereas rudd occupied the lowest trophic position (Table 1; Fig 2; *p* < 0.001 for all pairwise comparisons). The species also showed significant differences in their long-term use of littoral *versus* pelagic food resources in Milada (F_2,423_ = 525.8, *p* < 0.001), but not in Most (F_2,336_ = 2.00, *p* = 0.137). In Milada, rudd relied the most whereas perch relied the least on littoral food resources (Table 1; Fig 2; *p* < 0.001 for all pairwise comparisons).

**Table 1:** Mean (SD) and range of trophic position (TP) and littoral reliance (LR) estimates of the generalist fish populations in macrophyte-rich Milada and macrophyte-poor Most.

	Milada			Most			
	n	Mean (SD)	Range	n	Mean (SD)	Range	Statistics
*TP*							
Perch	167	4.64 (0.48)	3.23–5.93	159	3.79 (0.33)	3.02–4.49	t = 18.93; df = 292.82; ***p* < 0.001**
Roach	183	4.23 (0.32)	3.15–5.14	122	3.65 (0.35)	2.54–4.62	t = 14.82; df = 244.19; ***p* < 0.001**
Rudd	76	3.54 (0.39)	2.65–4.20	58	3.18 (0.30)	2.37–4.05	W = 3,305; n = 134; ***p* < 0.001**
*LR*							
Perch	167	0.39 (0.07)	0.22–0.63	159	0.48 (0.17)	0.10–0.86	W = 8,209; n = 326; ***p* < 0.001**
Roach	183	0.42 (0.07)	0.28–0.79	122	0.53 (0.24)	0.04–0.97	W = 7,276; n = 305; ***p* < 0.001**
Rudd	76	0.70 (0.08)	0.47–0.86	58	0.49 (0.12)	0.18–0.69	W = 4,164; n = 134; ***p* < 0.001**

Sample sizes (n) and results from the statistical tests are shown, with significant between-lake differences (*p* < 0.05) marked in bold.

### Between-lake differences in food webs and intraspecific niche variation

Perch collected for SIA were on average smaller (W = 10,167; n = 326; *p* < 0.001) whereas rudd were on average larger (W = 3,476; n = 134; *p* < 0.001) in Milada as compared to Most. No significant between-lake differences were observed in roach size (S2 Table).

All the three generalist fishes, but perch in particular, occupied a significantly higher trophic position in the macrophyte-rich Milada than in the macrophyte-poor Most (Table 1). Rudd relied significantly more whereas perch and roach relied less on littoral food resources in Milada than in Most (Table 1), likely due to prevalent herbivory by rudd and prevalent zooplanktivory by perch and rudd in Milada as compared to Most.

The isotopic niche widths estimated for the three generalist fishes differed significantly between the macrophyte-rich Milada and the macrophyte-poor Most. Perch and rudd showed significantly higher individual variation (*i*.*e*., variance) in trophic position in Milada than in Most, whereas no between-lake differences were observed for roach (Table 2; Fig 2).

**Table 2:** Individual niche variation within the generalist fish populations in macrophyte-rich Milada and macrophyte-poor Most, measured as the variance in trophic position (TP) and littoral reliance (LR) estimates.

		Variance		
	n	Milada	Most	Levene’s test
*TP*				
Perch	326	0.228	0.104	F_1,324_ = 19.563; ***p*< 0.001**
Roach	305	0.104	0.122	F_1,303_ = 152; *p* = 0.219
Rudd	134	0.152	0.092	F_1,132_ = 4.377; ***p* = 0.038**
*LR*				
Perch	326	0.0050	0.0283	F_1,324_ = 80.323; ***p* < 0.001**
Roach	305	0.0048	0.0576	F_1,303_ = 102.450; ***p* < 0.001**
Rudd	134	0.0063	0.0137	F_1,132_ = 13.892; ***p* < 0.001**

Results from the Levene’s test of equality of variances are shown, with significant between-lake differences (*p* < 0.05) marked in bold.

All the three species showed lower individual variation in the relative littoral reliance in macrophyte-rich Milada as compared to macrophyte-poor Most, with some individuals using mainly littoral benthic (*LR* > 0.5) and some mainly pelagic planktonic food resources (*LR* < 0.5) (Table 1; Fig 2). The variance tests were repeated after fixing the ranges in fish standard length between the lakes (*i*.*e*., after excluding exceptionally small and large individuals) but this did not change the results.

## Discussion

Our study demonstrates how habitat complexity, in terms of macrophyte vegetation, can influence the degree of individual niche variation within and resource competition between coexisting generalist species, but also how generalists with different feeding strategies can respond differently to differences in niche availability. As hypothesized, the three generalist species showed a higher intra-specific niche variation and a more distinct inter-specific niche segregation in the presence of abundant macrophyte vegetation. In essence, the generally higher trophic position of generalists suggests a more complex food-web structure (*i*.*e*., a longer food chain) in the macrophyte-rich Milada as compared to the macrophyte-poor Most. Perch and rudd showed higher individual variation in trophic position in Milada than in Most, apparently due to high trophic positions of some specialized piscivorous perch and low trophic positions of some specialized herbivorous rudd. At the same time, roach with limited potential to piscivorous or herbivorous specialization showed minor response to between-lake differences in macrophyte vegetation. Contrary to trophic position, the generalists in Milada showed less individual variation in littoral *versus* pelagic resource use than the conspecifics in Most, possibly due to a general paucity of preferred food resources in the latter ecosystem. Overall, our study gives further insights into the fundamental role of macrophytes in shaping the structure and function of lake food webs via their effects on trophic niche variation among generalist fishes.

The results from stable isotope analyses support our first hypothesis predicting significant long-term trophic niche segregation between the three generalist species. Perch, roach and rudd are all generalist species, but they show markedly different ontogenetic trajectories, with perch shifting diet from zooplankton to benthic macroinvertebrates and finally to fish [46,47] and rudd commonly shifting diet from zooplankton to benthic macroinvertebrates and finally to macrophytes [23,36]. Roach typically prefer zooplankton diet throughout ontogenesis, but may shift to benthic macroinvertebrates and plant matter if the zooplankton resources are scarce or absent [48]. The present study supports this conception of niche segregation between the three generalists as perch generally occupied the highest whereas rudd occupied the lowest trophic position. Herbivory is particularly common among rudd and hence this foraging strategy likely reduces resource competition with the more zooplanktivorous and benthivorous roach [16,49]. In the present study, roach and rudd showed significant differences in relative littoral reliance only in Milada. There, the abundant macrophyte vegetation apparently provide a profitable food resource for herbivorous rudd, but also a predation shelter for large-sized zooplankton, which was one of the main prey items for roach (S3 Table), as observed elsewhere [49,50]. Hence, our results suggest that macrophyte vegetation can reduce niche overlap, and hence interspecific resource competition, between coexisting generalist species [33,51,52]. The three generalist species likely compete for zooplankton resources at early life-stages, but the niche overlap apparently decreases with the individuals’ age and size due to increased piscivory by perch [21,33] and herbivory by rudd [53]. At the same time, piscivory by large perch generates novel intra- and inter-specific interactions as these individuals can predate upon smaller conspecifics as well as upon early life stages of other, potentially competitive fish species [21,33,46,54].

Macrophyte vegetation increases the overall physical complexity of the lake littoral zone, but also affects competitive and predatory interactions between different species and ontogenetic stages of fish [18,33,55]. Moreover, macrophytes can have direct effects on the lake total production via both increased primary production and increased density of benthic macroinvertebrates [15], large-sized zooplankton [12] as well as juvenile fish [56], but also indirectly via reducing resuspension of nutrients and fine particles from the sediment [13,57,58]. The effects of macrophytes on physical and biological complexity of the littoral zone can ultimately shape the overall structure and function of lake food webs [15,59]. While most studies have focused on macrophyte effects on competitive and/or predator–prey interactions [13,15,33], there has been limited empirical evidence of how macrophytes affect individual specialization among generalist species. The present study hence gives novel insights into how macrophyte vegetation can influence trophic niche variation within, as well as inter-specific resource competition between, generalist fishes with different foraging strategies. In particular, we found elevated and more variable trophic position of generalists in Milada, suggesting increased food-web complexity under the presence of macrophytes. This perception is supported by previous studies indicating that food-chain length generally increases with increasing resource availability [60,61], and it also supports our second hypothesis predicting higher intraspecific niche variation in the macrophyte-rich Milada. Our results also demonstrate clear between-lake differences in use of littoral and pelagic food resources, with generalists showing markedly larger individual variation in the macrophyte-poor Most. This high individual variation in littoral and pelagic resource use of generalists might indicate increased intra-specific competition due to a general paucity of food, forcing individuals to specialized foraging. This argument is consistent with the theory of trophic adaptability [62] and supported by previous studies from estuarine fishes where a scarcity of preferred food resources has led to population-level niche expansion [63]. This could explain why for instance some roach in Most have apparently specialized to zooplanktivory (*LR* < 0.5) and some other individuals have specialized to herbivory (*LR* > 0.5) instead of utilizing generalist diet consisting largely of preferred, large-sized benthic macroinvertebrates and terrestrial insects.

Our study demonstrates the fundamental role of habitat complexity (*i*.*e*., the presence/absence of macrophyte vegetation) in shaping individual variation in and resource use of generalist fishes. Such individual- and population-level differences in trophic niches of generalists can have complex effects on the community composition, through top-down and bottom-up trophic cascades, as well as on the entire ecosystems through altered energy-flow pathways and habitat coupling [5,7,64,65]. For example, the partial population-level niche specialization by herbivorous rudd, zooplanktivorous roach and piscivorous perch suggests high predation pressure on lower trophic levels and also limited habitat coupling by the species in the macrophyte-rich Milada. However, besides habitat (spatial) complexity, the intra- and inter-specific interactions within lake food webs can be fundamentally shaped by seasonal (temporal) variation in prey availability and environmental conditions [66]. Moreover, the other coexisting fishes, including piscivorous pike, catfish, pikeperch and zooplanktivorous whitefish, may have unexplored impacts on the niche width and resource use of the studied generalist species. Hence, seasonal studies and more controlled mesocosm experiments could give valuable insights into how macrophytes shape individual niche variation as well as intra- and inter-specific interactions among generalist fishes. Telemetry studies, in conjunction with trophic studies, could also provide important information about how macrophyte vegetation affects habitat use and activity levels of different generalist species and their ontogenetic stages, as well as individuals with different personalities [67].

## Supporting information

S1 Table Relative abundances (%) of the generalist species caught with survey gillnets from macrophyte-rich Milada and macrophyte-poor Most in 2013 and 2014. (PDF)

S2 Table Means (SD) and ranges of standard length (mm) and wet mass (g) of perch, roach and rudd sampled from macrophyte-rich Milada and macrophyte-poor Most in 2013–2014. (PDF)

S3 Table Relative proportions of main prey items in the gut contents of perch, roach and rudd caught from macrophyte-rich Milada and macrophyte-poor Most in 2013–2014. (PDF)

S4 Table Data for PONE-D-16-38804. (PDF)
