# Supplementary material for: Macrophytes shape trophic niche variation among generalist fishes
Source: PLoS One. 2017 May 9;12(5):e0177114. doi: 10.1371/journal.pone.0177114 (PMC5423621; doi:10.1371/journal.pone.0177114)
Supplement: S1 Table — (PDF) [file pone.0177114.s001.pdf]

S1 Table. Relative abundances (%) of the generalist species caught with survey gillnets from macrophyte-rich Milada and macrophyte-poor Most in 2013 and 2014.

	Milada		Most	
Species	2013	2014	2013	2014
Perch	53	66	51	72
Roach	44	32	42	24
Rudd	3	2	6	4
