# Supplementary material for: Macrophytes shape trophic niche variation among generalist fishes
Source: PLoS One. 2017 May 9;12(5):e0177114. doi: 10.1371/journal.pone.0177114 (PMC5423621; doi:10.1371/journal.pone.0177114)
Supplement: S2 Table — (PDF) [file pone.0177114.s002.pdf]

S2 Table. Means (SD) and ranges of standard length (mm) and wet mass (g) of perch, roach and rudd sampled from macrophyte-rich Milada and macrophyte-poor Most in 2013–2014.

			Standard length (mm)		Wet mass (g)	
Lake	Species	n	Mean (SD)	Range	Mean (SD)	Range
Milada	Perch	167	133 (54)	54–340	72 (111)	1–872
	Roach	183	157 (66)	48–320	139 (170)	1–787
	Rudd	76	228 (82)	43–355	476 (467)	1–1,574
Most	Perch	159	173 (89)	45–375	190 (271)	1–1,290
	Roach	122	164 (59)	51–270	135 (122)	2–476
	Rudd	58	152 (39)	51–235	97 (71)	2–306
