# Supplementary material for: Macrophytes shape trophic niche variation among generalist fishes
Source: PLoS One. 2017 May 9;12(5):e0177114. doi: 10.1371/journal.pone.0177114 (PMC5423621; doi:10.1371/journal.pone.0177114)
Supplement: S3 Table — (PDF) [file pone.0177114.s003.pdf]

S3 Table. Relative proportions of main prey items in the gut contents of perch, roach and rudd caught from macrophyte-rich Milada and macrophyte-poor Most in 2013–2014. Macroinvertebrates include littoral insect larvae, benthic crustaceans and molluscs, excluding the filtrate-feeding *Dreissena polymorpha*.

	Milada			Most		
Prey taxa	Perch	Roach	Rudd	Perch	Roach	Rudd
Zooplankton	0.67	0.51	0.00	0.34	0.13	0.00
Macroinvertebrates	0.13	0.24	0.08	0.31	0.02	0.04
D. polymorpha	0.00	0.04	0.00	0.00	0.60	0.00
Macrophytes	0.00	0.20	0.93	0.00	0.00	0.00
Periphyton	0.00	0.01	0.00	0.00	0.00	0.71
Detritus	0.00	0.00	0.00	0.00	0.25	0.25
Fish	0.20	0.00	0.00	0.35	0.00	0.00
