# Supplementary material for: Macrophytes shape trophic niche variation among generalist fishes
Source: PLoS One. 2017 May 9;12(5):e0177114. doi: 10.1371/journal.pone.0177114 (PMC5423621; doi:10.1371/journal.pone.0177114)
Supplement: S4 Table — (PDF) [file pone.0177114.s004.pdf]

##
READ ME
This file includes original data used and published in manuscript:
PONE-D-16-38804: 'Macrophytes shape trophic niche variation among generalist fishes'
The data is owned by the authors and provided as "S4 Table. Data for PONE-D-16-38804"

The data include following information:
"Lake" = Study lakes Milada and Most, both situated in northern Czech Republic
"Year" = Year of sampling
"Species" = common names of fishes;
--> perch Perca fluviatilis, roach Rutilus rutilus, rudd Scardinius erythrophthalmus
"SL_mm" = standard length in millimeters
"d13C" = $\delta^{13}C$ value of the fish muscle tissue
"d15N" = $\delta^{15}N$ value of the fish muscle tissue
"CN_ratio" = ratio of the carbon and nitrogen elements in the sample
"LR" = relative reliance of fish on littoral carbon sources
"TP" = relative trophic position of fish
See details of LR and TP: Post 2002; doi: 10.1890/0012-9658(2002)083[0703:USITET]2.0.CO;2
##

Lake	Year	Species	SL_mm	d13C	d15N	CN_ratio	LR	TP
Milada	2013	perch	59	-22.46	25.79	3.32	0.49	4.25
Milada	2013	perch	65	-22.96	26.82	3.3	0.46	4.58
Milada	2013	perch	68	-24.98	25.72	3.51	0.34	4.38
Milada	2013	perch	69	-22.87	26.18	3.37	0.47	4.39
Milada	2013	perch	70	-24.57	25.65	3.33	0.37	4.33
Milada	2013	perch	72	-24.98	25.48	3.33	0.34	4.31
Milada	2013	perch	77	-25.7	25.44	3.43	0.3	4.34
Milada	2013	perch	80	-23.01	25.96	3.28	0.46	4.33
Milada	2013	perch	90	-24.09	25.18	3.27	0.4	4.17
Milada	2013	perch	95	-22.29	25.97	3.3	0.5	4.29
Milada	2013	perch	97	-24.99	25.03	3.3	0.34	4.18
Milada	2013	perch	100	-24.86	23.94	3.31	0.35	3.85
Milada	2013	perch	100	-24.53	25.32	3.27	0.37	4.24
Milada	2013	perch	102	-24.47	31.01	3.3	0.37	5.91
Milada	2013	perch	105	-25.35	26.15	3.3	0.32	4.53
Milada	2013	perch	110	-24.6	25.33	3.3	0.37	4.24
Milada	2013	perch	110	-25.37	26.66	3.3	0.32	4.68
Milada	2013	perch	110	-24.74	27.03	3.28	0.36	4.75
Milada	2013	perch	110	-25.68	25.61	3.31	0.3	4.39
Milada	2013	perch	115	-25.48	26.01	3.29	0.31	4.49
Milada	2013	perch	115	-26.13	26.36	3.29	0.27	4.64
Milada	2013	perch	120	-23.51	26.62	3.25	0.43	4.55
Milada	2013	perch	120	-24.45	25.73	3.26	0.37	4.35
Milada	2013	perch	125	-24.24	26.31	3.28	0.39	4.51
Milada	2013	perch	130	-23.44	26.63	3.29	0.43	4.55
Milada	2013	perch	130	-23.46	26.66	3.27	0.43	4.56
Milada	2013	perch	130	-25.16	26.52	3.31	0.33	4.63
Milada	2013	perch	130	-25.3	26.32	3.24	0.32	4.58
Milada	2013	perch	135	-25.51	25.21	3.28	0.31	4.26

Milada	2013	perch	135	-23.45	27.25	3.23	0.43	4.74
Milada	2013	perch	140	-23.13	27.5	3.23	0.45	4.79
Milada	2013	perch	140	-22.64	26.41	3.24	0.48	4.44
Milada	2013	perch	140	-25.52	26.55	3.3	0.31	4.66
Milada	2013	perch	140	-23.35	26.52	3.28	0.44	4.52
Milada	2013	perch	145	-25.63	26.17	3.23	0.3	4.55
Milada	2013	perch	145	-25.61	25.99	3.3	0.31	4.5
Milada	2013	perch	150	-26.3	25.56	3.27	0.27	4.41
Milada	2013	perch	155	-24	22.02	3.28	0.4	3.23
Milada	2013	perch	155	-24.66	26.7	3.19	0.36	4.65
Milada	2013	perch	155	-26.02	26.3	3.24	0.28	4.61
Milada	2013	perch	155	-26.04	26.4	3.28	0.28	4.64
Milada	2013	perch	155	-23.04	26.56	3.28	0.46	4.51
Milada	2013	perch	155	-24.34	25.8	3.26	0.38	4.37
Milada	2013	perch	160	-24.59	26.19	3.25	0.37	4.49
Milada	2013	perch	160	-25.95	25.67	3.25	0.29	4.42
Milada	2013	perch	160	-25.76	25.69	3.24	0.3	4.42
Milada	2013	perch	165	-24.96	26.28	3.25	0.34	4.54
Milada	2013	perch	165	-24.43	27.35	3.2	0.38	4.83
Milada	2013	perch	165	-25.32	26.14	3.25	0.32	4.52
Milada	2013	perch	165	-25.05	25.96	3.21	0.34	4.45
Milada	2013	perch	165	-23.19	26.63	3.27	0.45	4.54
Milada	2013	perch	165	-21.45	26.61	3.28	0.55	4.43
Milada	2013	perch	175	-24.42	24.41	3.27	0.38	3.96
Milada	2013	perch	190	-26.12	26.39	3.24	0.28	4.64
Milada	2013	perch	190	-23.67	27	3.25	0.42	4.68
Milada	2013	perch	195	-25.64	26.33	3.19	0.3	4.6
Milada	2013	perch	200	-25.7	26.7	3.23	0.3	4.71
Milada	2013	perch	200	-23.67	26.62	3.24	0.42	4.56
Milada	2013	perch	205	-24.18	27.06	3.27	0.39	4.73
Milada	2013	perch	210	-25.38	26.25	3.23	0.32	4.56
Milada	2013	perch	210	-22.49	26.65	3.21	0.49	4.5
Milada	2013	perch	225	-24.57	26.6	3.23	0.37	4.61
Milada	2013	perch	230	-24.88	26	3.49	0.35	4.46
Milada	2013	perch	230	-25.88	26.7	3.26	0.29	4.72
Milada	2013	perch	240	-25.66	26.91	3.24	0.3	4.77
Milada	2013	perch	240	-25.95	26.98	3.24	0.29	4.81
Milada	2013	perch	245	-24.06	26.78	3.29	0.4	4.63
Milada	2013	perch	320	-24	26.45	3.24	0.4	4.53
Milada	2013	perch	340	-25.7	27	3.19	0.3	4.8
Milada	2013	perch	98	-24.63	25.95	3.37	0.36	4.42
Milada	2013	perch	100	-25.13	25.75	3.21	0.33	4.4
Milada	2013	perch	100	-24.04	25.58	3.37	0.4	4.28
Milada	2013	perch	101	-24.22	25.78	3.37	0.39	4.35
Milada	2013	roach	85	-23.9	24.64	3.3	0.41	4
Milada	2013	roach	90	-24.2	24.93	3.32	0.39	4.1
Milada	2013	roach	90	-21.51	25.6	3.3	0.55	4.14
Milada	2013	roach	93	-24.66	21.62	3	0.36	3.15
Milada	2013	roach	93	-24.48	24.54	3.34	0.37	4
Milada	2013	roach	95	-24.72	25.2	3.3	0.36	4.21

Milada	2013	roach	95	-20.64	24.51	3.34	0.6	3.76
Milada	2013	roach	98	-24.72	25.52	3.35	0.36	4.31
Milada	2013	roach	100	-24.83	25.69	3.39	0.35	4.36
Milada	2013	roach	100	-22.7	24.76	3.3	0.48	3.96
Milada	2013	roach	100	-25.05	24.59	3.32	0.34	4.05
Milada	2013	roach	100	-25.14	24.62	3.3	0.33	4.07
Milada	2013	roach	105	-22.76	24.12	3.78	0.47	3.77
Milada	2013	roach	105	-24.05	23.81	3.31	0.4	3.76
Milada	2013	roach	105	-22.56	24.8	2.9	0.49	3.96
Milada	2013	roach	105	-23.58	26.49	3.43	0.43	4.52
Milada	2013	roach	105	-24.61	24.26	3.34	0.37	3.93
Milada	2013	roach	105	-23.71	24.73	3.27	0.42	4.01
Milada	2013	roach	110	-23.92	24.8	3.2	0.41	4.04
Milada	2013	roach	110	-23.2	25.14	3.63	0.45	4.1
Milada	2013	roach	110	-25.05	25.05	3.35	0.34	4.19
Milada	2013	roach	110	-24.01	25.31	3.39	0.4	4.2
Milada	2013	roach	110	-24.65	24.69	3.27	0.36	4.06
Milada	2013	roach	110	-17.52	25.31	3.71	0.79	3.81
Milada	2013	roach	110	-24.16	24.49	3.32	0.39	3.97
Milada	2013	roach	115	-24.16	24.92	3.29	0.39	4.09
Milada	2013	roach	120	-23.2	25.05	3.26	0.45	4.08
Milada	2013	roach	125	-22.95	26.51	3.32	0.46	4.49
Milada	2013	roach	140	-23.12	26.73	3.33	0.45	4.56
Milada	2013	roach	150	-22.57	26.19	3.28	0.49	4.37
Milada	2013	roach	150	-23.94	24.85	3.17	0.4	4.06
Milada	2013	roach	155	-23.42	25.21	3.19	0.44	4.14
Milada	2013	roach	155	-21.69	25.38	3.29	0.54	4.08
Milada	2013	roach	155	-24.28	21.72	3.22	0.38	3.16
Milada	2013	roach	155	-22.85	26.84	3.3	0.47	4.58
Milada	2013	roach	155	-23.5	25.58	3.31	0.43	4.25
Milada	2013	roach	155	-22.8	25.79	3.29	0.47	4.27
Milada	2013	roach	160	-23.6	24.57	3.21	0.42	3.96
Milada	2013	roach	160	-22.68	25.39	3.27	0.48	4.14
Milada	2013	roach	160	-24.08	24.09	3.66	0.4	3.85
Milada	2013	roach	160	-21.94	25.87	3.24	0.52	4.24
Milada	2013	roach	160	-24.73	26.96	3.41	0.36	4.73
Milada	2013	roach	165	-21.23	25.49	3.24	0.57	4.09
Milada	2013	roach	165	-23.61	26.03	3.55	0.42	4.39
Milada	2013	roach	165	-24.76	25.15	3.29	0.36	4.2
Milada	2013	roach	170	-23.9	26.6	3.28	0.41	4.57
Milada	2013	roach	170	-22	26.13	3.21	0.52	4.32
Milada	2013	roach	170	-24.93	24.95	3.33	0.35	4.15
Milada	2013	roach	170	-24.96	24.36	3.25	0.34	3.98
Milada	2013	roach	175	-24.19	25.12	3.36	0.39	4.15
Milada	2013	roach	175	-22.53	25.96	3.27	0.49	4.3
Milada	2013	roach	175	-22.9	25.44	3.28	0.47	4.17
Milada	2013	roach	175	-23.03	27.07	3.29	0.46	4.66
Milada	2013	roach	180	-24.01	26.92	3.18	0.4	4.67
Milada	2013	roach	180	-24.31	25.67	3.29	0.38	4.32
Milada	2013	roach	180	-24.65	24.21	3.21	0.36	3.92

Milada	2013	roach	185	-23.57	26.45	3.27	0.43	4.51
Milada	2013	roach	185	-22.29	26.09	3.29	0.5	4.33
Milada	2013	roach	185	-21.98	26.28	3.75	0.52	4.36
Milada	2013	roach	185	-23.7	25.09	3.32	0.42	4.12
Milada	2013	roach	190	-23.57	24.86	2.95	0.43	4.04
Milada	2013	roach	190	-25.82	24.49	3.32	0.29	4.07
Milada	2013	roach	195	-23.2	25.52	3.71	0.45	4.21
Milada	2013	roach	195	-23.62	24.63	3.43	0.42	3.98
Milada	2013	roach	195	-22.2	26.08	3.27	0.51	4.32
Milada	2013	roach	195	-23.43	25.06	3.26	0.44	4.09
Milada	2013	roach	200	-24.22	25.68	3.35	0.39	4.32
Milada	2013	roach	200	-22.82	25.26	3.21	0.47	4.12
Milada	2013	roach	205	-25.05	24.85	3.27	0.34	4.13
Milada	2013	roach	205	-24.56	26.35	3.28	0.37	4.54
Milada	2013	roach	210	-23.3	25.85	2.87	0.44	4.32
Milada	2013	roach	215	-24	23.98	3.35	0.4	3.81
Milada	2013	roach	215	-24.16	27.77	3.25	0.39	4.93
Milada	2013	roach	240	-23.74	25.16	3.3	0.42	4.14
Milada	2013	roach	240	-24.68	24.49	3.3	0.36	4
Milada	2013	roach	250	-24.73	24.88	3.34	0.36	4.12
Milada	2013	roach	250	-23.28	26.8	3.35	0.44	4.59
Milada	2013	roach	250	-24.12	26.9	3.26	0.39	4.68
Milada	2013	roach	260	-23.66	25.55	3.48	0.42	4.25
Milada	2013	roach	260	-25.02	24.68	3.28	0.34	4.08
Milada	2013	roach	260	-24.79	24.78	3.34	0.35	4.09
Milada	2013	roach	265	-24.25	25.18	3.33	0.39	4.18
Milada	2013	roach	275	-24.17	24.48	3.35	0.39	3.97
Milada	2013	roach	275	-23.72	25.58	3.44	0.42	4.26
Milada	2013	roach	285	-24.64	25.38	3.72	0.36	4.26
Milada	2013	roach	285	-25.21	24.54	3.33	0.33	4.05
Milada	2013	roach	320	-24.89	24.99	3.34	0.35	4.16
Milada	2013	roach	105	-24.64	25.66	3.34	0.36	4.34
Milada	2013	roach	108	-23.71	24.78	3.21	0.42	4.03
Milada	2013	roach	110	-25.44	24.42	3.29	0.32	4.02
Milada	2013	roach	60	-25.55	24.68	3.35	0.31	4.11
Milada	2013	roach	60	-25.87	24.82	3.35	0.29	4.17
Milada	2013	roach	65	-26.02	25.01	3.11	0.28	4.23
Milada	2013	roach	70	-25.72	25.25	3.54	0.3	4.28
Milada	2013	roach	70	-25.7	25.03	3.43	0.3	4.22
Milada	2013	roach	85	-18.44	24.18	3.28	0.73	3.53
Milada	2013	rudd	95	-21.63	24	3.18	0.54	3.67
Milada	2013	rudd	110	-21.79	23.81	3.27	0.53	3.62
Milada	2013	rudd	155	-21.26	22.07	3.24	0.56	3.08
Milada	2013	rudd	160	-20.55	22.82	3.39	0.61	3.26
Milada	2013	rudd	160	-18.14	21.83	3.26	0.75	2.82
Milada	2013	rudd	160	-18.1	22.24	3.29	0.75	2.94
Milada	2013	rudd	165	-20.34	23.18	3.24	0.62	3.35
Milada	2013	rudd	165	-18.13	22.2	3.26	0.75	2.93
Milada	2013	rudd	170	-18.58	22.13	3.27	0.72	2.94
Milada	2013	rudd	170	-17.67	22.55	3.18	0.78	3.01

Milada	2013	rudd	175	-17.52	22.25	3.24	0.79	2.91
Milada	2013	rudd	175	-18.56	21.82	3.27	0.72	2.84
Milada	2013	rudd	180	-16.43	24.89	3.2	0.85	3.62
Milada	2013	rudd	180	-18.85	21.09	3.55	0.71	2.65
Milada	2013	rudd	185	-16.99	22.23	3.2	0.82	2.87
Milada	2013	rudd	185	-16.78	23.2	3.26	0.83	3.14
Milada	2013	rudd	190	-16.23	23.03	3.2	0.86	3.06
Milada	2013	rudd	195	-19.46	22.48	3.26	0.67	3.09
Milada	2013	rudd	200	-18.69	25	3.24	0.72	3.79
Milada	2013	rudd	200	-18.5	21.89	3.27	0.73	2.86
Milada	2013	rudd	210	-17.04	23.06	3.24	0.81	3.12
Milada	2013	rudd	210	-17.93	22.1	3.27	0.76	2.89
Milada	2013	rudd	220	-17.84	25.05	3.26	0.77	3.75
Milada	2013	rudd	220	-19.32	22.26	3.27	0.68	3.02
Milada	2013	rudd	225	-18.73	23.53	3.46	0.71	3.36
Milada	2013	rudd	235	-18.95	23.72	3.25	0.7	3.43
Milada	2013	rudd	235	-18.3	22.06	3.24	0.74	2.9
Milada	2013	rudd	240	-18.84	24.32	3.24	0.71	3.6
Milada	2013	rudd	255	-19.43	23.73	3.27	0.67	3.46
Milada	2013	rudd	285	-20.14	24.08	3.24	0.63	3.6
Milada	2013	rudd	290	-18.63	24.38	3.51	0.72	3.6
Milada	2013	rudd	295	-19.62	24.48	3.3	0.66	3.69
Milada	2013	rudd	305	-19.3	24.58	3.3	0.68	3.7
Milada	2013	rudd	310	-19.45	24.37	3.24	0.67	3.65
Milada	2013	rudd	320	-19.32	24.21	3.37	0.68	3.59
Milada	2013	rudd	325	-19.24	24.72	3.43	0.68	3.74
Milada	2013	rudd	330	-19.47	24.38	3.28	0.67	3.65
Milada	2013	rudd	340	-19.32	25.05	2.96	0.68	3.84
Milada	2013	rudd	340	-20.21	25.02	3.32	0.63	3.88
Milada	2013	rudd	350	-20.25	25.48	3.33	0.62	4.02
Milada	2013	rudd	350	-20.11	25.11	3.34	0.63	3.91
Milada	2014	perch	75	-23.45	24.84	3.35	0.43	4.03
Milada	2014	perch	90	-23.92	29.11	3.3	0.41	5.31
Milada	2014	perch	95	-23.44	27.66	3.33	0.43	4.86
Milada	2014	perch	110	-24.95	26.93	3.34	0.34	4.73
Milada	2014	perch	110	-25.1	28.25	3.31	0.34	5.13
Milada	2014	perch	115	-25.2	27.2	3.26	0.33	4.83
Milada	2014	perch	120	-22.97	28.57	3.32	0.46	5.1
Milada	2014	perch	135	-22.77	29.17	3.26	0.47	5.26
Milada	2014	perch	165	-23.21	29.68	3.19	0.45	5.44
Milada	2014	perch	175	-23.5	28.62	3.23	0.43	5.14
Milada	2014	perch	130	-23.74	29.53	3.27	0.42	5.43
Milada	2014	perch	140	-24.35	29.93	3.27	0.38	5.58
Milada	2014	perch	165	-22.42	28.74	3.2	0.49	5.11
Milada	2014	perch	150	-22.97	28.9	3.27	0.46	5.19
Milada	2014	perch	155	-23.06	29.18	3.29	0.46	5.28
Milada	2014	perch	175	-23.24	28.83	3.27	0.45	5.19
Milada	2014	perch	62	-23.65	24.92	3.42	0.42	4.06
Milada	2014	perch	63	-23.04	24.62	3.42	0.46	3.94
Milada	2014	perch	66	-23.72	24.93	3.36	0.42	4.07

Milada	2014	perch	69	-22.3	24.53	3.35	0.5	3.87
Milada	2014	perch	70	-23.24	24.62	3.46	0.45	3.95
Milada	2014	perch	78	-23.17	24.91	3.37	0.45	4.03
Milada	2014	perch	100	-25.03	28.77	3.29	0.34	5.28
Milada	2014	perch	110	-24.79	27.93	3.28	0.35	5.02
Milada	2014	perch	115	-24.7	27.22	3.29	0.36	4.81
Milada	2014	perch	130	-23.98	27.88	3.27	0.4	4.96
Milada	2014	perch	155	-23.73	26.34	3.28	0.42	4.48
Milada	2014	perch	170	-22.33	29.06	3.2	0.5	5.2
Milada	2014	perch	170	-23.83	28.09	3.21	0.41	5.01
Milada	2014	perch	185	-22.68	29.8	3.23	0.48	5.44
Milada	2014	perch	190	-23.67	27.78	3.2	0.42	4.91
Milada	2014	perch	205	-24.2	28.64	3.22	0.39	5.19
Milada	2014	perch	215	-24.03	27.68	3.22	0.4	4.9
Milada	2014	perch	220	-24.1	27.71	3.2	0.4	4.91
Milada	2014	perch	215	-23.73	28.35	3.25	0.42	5.08
Milada	2014	perch	260	-22.89	29.17	3.25	0.47	5.27
Milada	2014	perch	71	-23.55	25.15	3.3	0.43	4.12
Milada	2014	perch	72	-23.86	25.09	3.3	0.41	4.13
Milada	2014	perch	73	-23.11	24.53	3.35	0.45	3.92
Milada	2014	perch	100	-25.14	28.01	3.29	0.33	5.06
Milada	2014	perch	120	-25.28	27.84	3.29	0.33	5.02
Milada	2014	perch	110	-25.18	27.89	3.35	0.33	5.03
Milada	2014	perch	135	-26.06	28.3	3.27	0.28	5.2
Milada	2014	perch	160	-25.39	31.02	3.25	0.32	5.96
Milada	2014	perch	220	-24.78	28.13	3.25	0.36	5.08
Milada	2014	perch	155	-23.2	28.88	3.28	0.45	5.2
Milada	2014	perch	65	-22.81	24.7	3.35	0.47	3.95
Milada	2014	perch	72	-23.86	24.83	3.3	0.41	4.05
Milada	2014	perch	105	-25.28	26.78	3.25	0.33	4.71
Milada	2014	perch	55	-21.7	24.13	3.39	0.54	3.71
Milada	2014	perch	100	-25.6	28.67	3.28	0.31	5.28
Milada	2014	perch	105	-24.96	26.45	3.25	0.34	4.59
Milada	2014	perch	57	-20.22	25.58	3.32	0.63	4.05
Milada	2014	perch	59	-22.56	23.9	3.34	0.49	3.7
Milada	2014	perch	62	-22.3	24.85	3.45	0.5	3.96
Milada	2014	perch	63	-20.72	24.61	3.37	0.6	3.79
Milada	2014	perch	70	-23.18	25.2	3.34	0.45	4.12
Milada	2014	perch	140	-23.36	28.29	3.27	0.44	5.04
Milada	2014	perch	165	-23.18	27.53	3.23	0.45	4.8
Milada	2014	perch	185	-23.43	27.71	3.25	0.43	4.87
Milada	2014	perch	210	-23.48	28.6	3.25	0.43	5.14
Milada	2014	perch	95	-23.79	26.23	3.33	0.41	4.46
Milada	2014	perch	125	-24.07	27.12	3.32	0.4	4.74
Milada	2014	perch	160	-23.94	28.08	3.28	0.4	5.01
Milada	2014	perch	80	-23.09	24.7	3.36	0.46	3.97
Milada	2014	perch	120	-23.81	27.8	3.23	0.41	4.92
Milada	2014	perch	125	-24.89	27.87	3.27	0.35	5.01
Milada	2014	perch	165	-25.15	28.91	3.26	0.33	5.33
Milada	2014	perch	280	-23.61	28.4	3.3	0.42	5.08

Milada	2014	perch	54	-23.08	25.21	3.36	0.46	4.12
Milada	2014	perch	61	-22.63	24.19	3.42	0.48	3.79
Milada	2014	perch	69	-22.86	24.66	3.35	0.47	3.94
Milada	2014	perch	76	-23.46	24.47	3.33	0.43	3.92
Milada	2014	perch	78	-24.41	27.34	3.3	0.38	4.82
Milada	2014	perch	84	-23.32	24.54	3.39	0.44	3.93
Milada	2014	perch	95	-24.56	26.87	3.27	0.37	4.69
Milada	2014	perch	135	-25.15	27.36	3.27	0.33	4.87
Milada	2014	perch	145	-24.96	28.26	3.24	0.34	5.13
Milada	2014	perch	145	-25.09	28.02	3.25	0.34	5.06
Milada	2014	perch	150	-24.98	28.78	3.27	0.34	5.28
Milada	2014	perch	150	-24.49	28.2	3.25	0.37	5.08
Milada	2014	perch	97	-23.25	26.64	3.29	0.45	4.55
Milada	2014	perch	165	-23.53	27.52	3.26	0.43	4.82
Milada	2014	perch	65	-21.86	25.43	3.36	0.53	4.11
Milada	2014	perch	68	-24.32	26.15	3.39	0.38	4.47
Milada	2014	perch	115	-27.13	29.13	3.28	0.22	5.51
Milada	2014	perch	115	-24.73	27.19	3.32	0.36	4.8
Milada	2014	perch	115	-25.74	28.14	3.39	0.3	5.14
Milada	2014	perch	125	-26.18	29.36	3.31	0.27	5.52
Milada	2014	perch	100	-24.87	27.64	3.34	0.35	4.94
Milada	2014	perch	110	-25.32	28.37	3.34	0.32	5.18
Milada	2014	perch	115	-25.66	29.07	3.37	0.3	5.41
Milada	2014	perch	230	-25.74	29.61	3.24	0.3	5.57
Milada	2014	perch	58	-22.48	24.05	3.36	0.49	3.74
Milada	2014	roach	52	-22.64	25.29	3.5	0.48	4.11
Milada	2014	roach	180	-23.77	27.38	3.51	0.41	4.8
Milada	2014	roach	54	-23.01	26	3.3	0.46	4.34
Milada	2014	roach	55	-21.58	24.97	3.43	0.54	3.95
Milada	2014	roach	72	-23.7	23.74	3.31	0.42	3.72
Milada	2014	roach	79	-23.7	23.86	3.52	0.42	3.75
Milada	2014	roach	85	-23.7	23.91	3.38	0.42	3.77
Milada	2014	roach	100	-23.51	26.16	3.33	0.43	4.42
Milada	2014	roach	160	-23.07	26.66	3.31	0.46	4.54
Milada	2014	roach	170	-22.86	24.83	3.64	0.47	3.99
Milada	2014	roach	230	-22.95	25.93	3.3	0.46	4.32
Milada	2014	roach	265	-24.17	27.08	3.29	0.39	4.73
Milada	2014	roach	225	-22.56	26.56	3.35	0.49	4.48
Milada	2014	roach	83	-22.07	25.59	3.39	0.52	4.17
Milada	2014	roach	110	-24.23	25.6	3.3	0.39	4.3
Milada	2014	roach	115	-23.64	25.44	3.4	0.42	4.21
Milada	2014	roach	115	-24.32	25.55	3.39	0.38	4.29
Milada	2014	roach	160	-24.53	25.98	3.38	0.37	4.43
Milada	2014	roach	170	-24.69	27.24	3.37	0.36	4.81
Milada	2014	roach	180	-23.13	26.95	3.31	0.45	4.63
Milada	2014	roach	230	-23.52	27.1	3.4	0.43	4.7
Milada	2014	roach	230	-24.44	26.42	3.37	0.38	4.55
Milada	2014	roach	73	-23.33	24.47	3.47	0.44	3.91
Milada	2014	roach	250	-24.77	27.11	3.47	0.36	4.77
Milada	2014	roach	290	-24.86	26.84	3.38	0.35	4.7

Milada	2014	roach	310	-24.58	26.3	3.33	0.37	4.52
Milada	2014	roach	175	-24.5	26.53	3.31	0.37	4.59
Milada	2014	roach	68	-23.28	23.57	3.45	0.44	3.65
Milada	2014	roach	120	-23.56	25.1	3.32	0.43	4.11
Milada	2014	roach	130	-23.11	25.11	3.37	0.45	4.09
Milada	2014	roach	150	-23.12	23.94	3.33	0.45	3.74
Milada	2014	roach	160	-24.17	25.98	3.29	0.39	4.41
Milada	2014	roach	165	-21.65	26.72	3.47	0.54	4.47
Milada	2014	roach	230	-23.18	25.7	3.31	0.45	4.27
Milada	2014	roach	210	-23.72	25.64	3.37	0.42	4.28
Milada	2014	roach	220	-23.27	26.95	3.44	0.44	4.64
Milada	2014	roach	240	-23.06	25.34	3.41	0.46	4.15
Milada	2014	roach	240	-24.95	26.53	3.4	0.34	4.62
Milada	2014	roach	275	-24.91	26.96	3.42	0.35	4.74
Milada	2014	roach	260	-25.72	26.93	4.24	0.3	4.78
Milada	2014	roach	120	-23.35	25.36	3.41	0.44	4.18
Milada	2014	roach	120	-23.89	25.56	3.54	0.41	4.27
Milada	2014	roach	130	-23.87	25.84	3.45	0.41	4.35
Milada	2014	roach	130	-23.44	24.61	3.47	0.43	3.96
Milada	2014	roach	130	-24.34	25.6	3.58	0.38	4.31
Milada	2014	roach	145	-23.57	25.57	3.46	0.43	4.25
Milada	2014	roach	150	-22.75	25.62	3.37	0.48	4.21
Milada	2014	roach	165	-20.83	25.57	3.42	0.59	4.08
Milada	2014	roach	165	-23.74	27.03	3.44	0.42	4.69
Milada	2014	roach	170	-22.27	26.82	3.47	0.5	4.54
Milada	2014	roach	80	-23.4	24.2	3.49	0.44	3.84
Milada	2014	roach	115	-24.03	26.32	3.4	0.4	4.5
Milada	2014	roach	120	-23.64	25.29	3.5	0.42	4.17
Milada	2014	roach	165	-23.54	26.56	3.7	0.43	4.54
Milada	2014	roach	60	-22.24	24.69	3.43	0.51	3.91
Milada	2014	roach	75	-23.22	23.57	3.45	0.45	3.64
Milada	2014	roach	112	-23.11	25.44	3.45	0.45	4.18
Milada	2014	roach	124	-22.6	25.57	3.35	0.48	4.19
Milada	2014	roach	128	-22.56	25.6	3.31	0.49	4.2
Milada	2014	roach	138	-23.59	25.89	3.29	0.43	4.34
Milada	2014	roach	160	-23.68	27.35	3.45	0.42	4.78
Milada	2014	roach	175	-23.89	28.53	3.33	0.41	5.14
Milada	2014	roach	183	-24.09	28.04	3.41	0.4	5.01
Milada	2014	roach	170	-22.94	26.37	3.36	0.46	4.45
Milada	2014	roach	210	-22.87	27.23	3.44	0.47	4.7
Milada	2014	roach	305	-24	26.68	3.43	0.4	4.6
Milada	2014	roach	163	-23.38	27.17	3.46	0.44	4.71
Milada	2014	roach	195	-24.7	25.71	3.42	0.36	4.36
Milada	2014	roach	237	-23.21	27.05	3.4	0.45	4.66
Milada	2014	roach	270	-25.18	26.64	3.56	0.33	4.66
Milada	2014	roach	295	-24.87	26.75	3.49	0.35	4.68
Milada	2014	roach	300	-24.58	26.47	3.46	0.37	4.58
Milada	2014	roach	320	-22.94	26.54	3.4	0.46	4.5
Milada	2014	roach	48	-21.98	24.96	3.53	0.52	3.97
Milada	2014	roach	105	-23.75	26.45	3.31	0.42	4.52

Milada	2014	roach	110	-23.65	25.76	3.47	0.42	4.31
Milada	2014	roach	110	-24.3	25.76	3.37	0.38	4.35
Milada	2014	roach	120	-25.4	22.94	3.31	0.32	3.59
Milada	2014	roach	55	-23.19	24.34	3.47	0.45	3.87
Milada	2014	roach	61	-23.69	24.14	3.52	0.42	3.84
Milada	2014	roach	63	-22.91	24.38	3.51	0.47	3.86
Milada	2014	roach	165	-23.91	26.91	3.56	0.41	4.67
Milada	2014	roach	70	-22.58	24.63	3.4	0.49	3.91
Milada	2014	roach	65	-23.28	24.25	3.41	0.44	3.84
Milada	2014	roach	115	-23.31	25.44	3.33	0.44	4.2
Milada	2014	roach	60	-22.79	24.65	3.42	0.47	3.93
Milada	2014	roach	70	-23.12	23.9	3.4	0.45	3.73
Milada	2014	rudd	125	-20.14	25.44	3.41	0.63	4.01
Milada	2014	rudd	135	-19.65	26.17	3.37	0.66	4.19
Milada	2014	rudd	145	-18.39	24.37	3.31	0.73	3.59
Milada	2014	rudd	150	-19.17	25.8	3.32	0.69	4.05
Milada	2014	rudd	110	-21.31	24.33	3.41	0.56	3.75
Milada	2014	rudd	145	-18.32	25.6	3.31	0.74	3.94
Milada	2014	rudd	185	-17.9	24.85	3.33	0.76	3.7
Milada	2014	rudd	215	-18.19	25.11	3.25	0.75	3.79
Milada	2014	rudd	43	-20.8	24.62	3.51	0.59	3.8
Milada	2014	rudd	43	-22.3	24.03	3.55	0.5	3.72
Milada	2014	rudd	45	-22.78	24	3.56	0.47	3.74
Milada	2014	rudd	160	-18.65	24.56	3.27	0.72	3.66
Milada	2014	rudd	235	-17.32	23.31	3.25	0.8	3.21
Milada	2014	rudd	235	-16.28	24.28	3.25	0.86	3.43
Milada	2014	rudd	250	-18.43	24.83	3.31	0.73	3.72
Milada	2014	rudd	355	-18.77	24.51	3.38	0.71	3.65
Milada	2014	rudd	225	-17.53	24.8	3.27	0.78	3.66
Milada	2014	rudd	320	-19.18	25.15	3.3	0.69	3.86
Milada	2014	rudd	340	-19.04	25.55	3.3	0.7	3.97
Milada	2014	rudd	350	-18.52	24.01	3.31	0.73	3.49
Milada	2014	rudd	225	-17.1	25.27	3.3	0.81	3.77
Milada	2014	rudd	325	-19.67	26.18	3.43	0.66	4.2
Milada	2014	rudd	220	-17.69	25.44	3.34	0.78	3.86
Milada	2014	rudd	245	-17.96	23.21	3.26	0.76	3.22
Milada	2014	rudd	340	-19.24	25.63	3.33	0.68	4.01
Milada	2014	rudd	305	-19.47	26.21	3.35	0.67	4.19
Milada	2014	rudd	320	-20.37	25.11	3.56	0.62	3.92
Milada	2014	rudd	325	-18.44	24.55	3.36	0.73	3.64
Milada	2014	rudd	330	-19.44	24.76	3.45	0.67	3.76
Milada	2014	rudd	340	-20.23	24.69	3.5	0.62	3.79
Milada	2014	rudd	350	-18.36	25.44	3.37	0.74	3.9
Milada	2014	rudd	320	-19.29	25.41	3.48	0.68	3.94
Milada	2014	rudd	213	-18.73	25.08	3.38	0.71	3.81
Milada	2014	rudd	230	-18.63	23.61	3.3	0.72	3.37
Milada	2014	rudd	150	-19.88	24.49	3.29	0.65	3.71
Most	2013	perch	360	-24	17.5	3.22	0.49	4.07
Most	2013	perch	125	-20.97	17.2	3.35	0.77	3.72
Most	2013	perch	170	-22.58	16.05	3.32	0.62	3.52

Most	2013	perch	70	-23.05	14.21	3.38	0.58	3.02
Most	2013	perch	175	-23.28	16.62	3.27	0.56	3.75
Most	2013	perch	125	-23.36	16.04	3.28	0.55	3.58
Most	2013	perch	80	-26.04	15.86	3.3	0.31	3.76
Most	2013	perch	76	-23.36	15.84	3.26	0.55	3.52
Most	2013	perch	70	-24.87	15.77	3.3	0.42	3.63
Most	2013	perch	300	-25.17	17.73	3.2	0.39	4.24
Most	2013	perch	305	-24.75	17.41	3.21	0.43	4.11
Most	2013	perch	130	-24.21	15.4	3.31	0.48	3.47
Most	2013	perch	200	-22.69	15.91	3.3	0.61	3.49
Most	2013	perch	210	-24.42	16.55	3.25	0.46	3.82
Most	2013	perch	160	-23.7	16.33	3.32	0.52	3.7
Most	2013	perch	165	-24.2	16.04	3.28	0.48	3.66
Most	2013	perch	310	-23.92	17.15	3.26	0.5	3.96
Most	2013	perch	280	-24.28	17.27	3.19	0.47	4.03
Most	2013	perch	215	-22.96	17.66	3.26	0.59	4.02
Most	2013	perch	135	-20.33	17.04	3.26	0.82	3.62
Most	2013	perch	115	-20.81	17.07	3.29	0.78	3.66
Most	2013	perch	125	-22.8	16.99	3.32	0.6	3.81
Most	2013	perch	140	-23.53	17.73	3.3	0.54	4.1
Most	2013	perch	140	-23.5	16.51	3.28	0.54	3.73
Most	2013	perch	145	-23.03	16.98	3.27	0.58	3.83
Most	2013	perch	150	-24.14	16.29	3.32	0.48	3.72
Most	2013	perch	235	-25.34	17.11	3.25	0.37	4.07
Most	2013	perch	205	-22.68	16.96	3.3	0.61	3.8
Most	2013	perch	230	-24.42	17.74	3.28	0.46	4.17
Most	2013	perch	255	-25.23	17.3	3.28	0.38	4.12
Most	2013	perch	345	-25.56	18.16	3.16	0.36	4.4
Most	2013	perch	295	-25.25	17.62	3.23	0.38	4.21
Most	2013	perch	315	-24.73	17.93	3.22	0.43	4.26
Most	2013	perch	160	-21.45	17.6	3.33	0.72	3.88
Most	2013	perch	295	-24.88	17.31	3.28	0.42	4.09
Most	2013	perch	310	-24.56	17.94	3.24	0.45	4.25
Most	2013	perch	295	-25.07	18.06	3.26	0.4	4.32
Most	2013	perch	270	-25.57	17.73	3.26	0.35	4.27
Most	2013	perch	300	-25.63	17.89	3.24	0.35	4.32
Most	2013	perch	280	-24.62	17.15	3.2	0.44	4.02
Most	2013	perch	145	-21.18	18.13	3.29	0.75	4.01
Most	2013	perch	130	-21.74	17.27	3.28	0.7	3.81
Most	2013	perch	135	-21.63	18.14	3.29	0.71	4.05
Most	2013	perch	220	-24.87	15.6	3.29	0.42	3.58
Most	2013	perch	240	-26.74	17.68	3.26	0.25	4.36
Most	2013	perch	205	-25.5	16.05	3.29	0.36	3.77
Most	2013	perch	170	-22.33	16.73	3.29	0.65	3.7
Most	2013	perch	165	-22.47	17.07	3.29	0.63	3.81
Most	2013	perch	170	-24.49	15.93	3.29	0.45	3.65
Most	2013	perch	170	-25.63	14.75	3.33	0.35	3.4
Most	2013	perch	170	-27.03	17.07	3.22	0.22	4.2
Most	2013	perch	145	-23.78	16.64	3.29	0.52	3.8
Most	2013	perch	310	-24.58	17.02	3.19	0.44	3.98

Most	2013	perch	255	-23.38	17.87	3.25	0.55	4.12
Most	2013	perch	265	-24.11	17.86	3.25	0.49	4.18
Most	2013	perch	215	-23.04	17.66	3.27	0.58	4.03
Most	2013	perch	225	-23.09	18.27	3.27	0.58	4.21
Most	2013	perch	145	-20.93	17.61	3.34	0.77	3.84
Most	2013	perch	335	-24.34	17.72	3.2	0.46	4.16
Most	2013	perch	160	-20.03	17.69	3.33	0.85	3.78
Most	2013	perch	145	-21.11	17.55	3.3	0.75	3.83
Most	2013	perch	140	-21.24	17.88	3.3	0.74	3.94
Most	2013	perch	160	-21.37	17.52	3.34	0.73	3.85
Most	2013	perch	155	-20.93	17.3	3.35	0.77	3.74
Most	2013	perch	135	-20.22	17.7	3.31	0.83	3.8
Most	2013	perch	135	-22.82	17.58	3.32	0.6	3.99
Most	2013	perch	140	-22.07	17.31	3.3	0.67	3.85
Most	2013	perch	140	-20.79	17.57	3.33	0.78	3.81
Most	2013	perch	140	-20.54	17.52	3.24	0.81	3.77
Most	2013	perch	130	-19.89	17.71	3.33	0.86	3.77
Most	2013	perch	330	-24.21	17.71	3.21	0.48	4.15
Most	2013	roach	225	-29.12	17.88	3.24	0.04	4.62
Most	2013	roach	135	-21.12	15.38	3.3	0.75	3.2
Most	2013	roach	250	-28.39	16.57	3.3	0.1	4.17
Most	2013	roach	130	-21.27	15.79	3.29	0.74	3.33
Most	2013	roach	200	-25.46	15.53	3.26	0.36	3.61
Most	2013	roach	250	-28.06	15.62	3.32	0.13	3.87
Most	2013	roach	240	-26.95	15.49	3.31	0.23	3.73
Most	2013	roach	230	-27.62	15.65	3.3	0.17	3.84
Most	2013	roach	210	-25.27	15.07	3.32	0.38	3.46
Most	2013	roach	240	-27.36	16.25	3.29	0.19	3.99
Most	2013	roach	230	-26.45	16.87	3.33	0.27	4.09
Most	2013	roach	215	-26.79	15.28	3.23	0.24	3.66
Most	2013	roach	235	-28.02	16.6	3.35	0.13	4.15
Most	2013	roach	125	-21.97	16.89	3.3	0.68	3.71
Most	2013	roach	120	-20.19	16.15	3.28	0.84	3.34
Most	2013	roach	125	-21.45	16.56	3.28	0.72	3.57
Most	2013	roach	105	-20.07	16.67	3.32	0.85	3.48
Most	2013	roach	215	-25.83	15.35	3.35	0.33	3.59
Most	2013	roach	210	-27.36	16.07	3.26	0.19	3.94
Most	2013	roach	245	-27.63	16.11	3.34	0.17	3.97
Most	2013	roach	135	-22.95	17.06	3.27	0.59	3.85
Most	2013	roach	205	-24.82	16.68	3.32	0.42	3.9
Most	2013	roach	120	-23.55	16.11	3.25	0.54	3.62
Most	2013	roach	130	-21.84	17.2	3.31	0.69	3.79
Most	2013	roach	120	-21.4	15.22	3.29	0.73	3.17
Most	2013	roach	125	-21.95	17.21	3.26	0.68	3.8
Most	2013	roach	130	-24.08	16.18	3.3	0.49	3.69
Most	2013	roach	120	-21.3	15.53	3.27	0.74	3.25
Most	2013	roach	130	-20.3	16.3	3.3	0.83	3.39
Most	2013	roach	135	-21.19	14.41	3.29	0.75	2.92
Most	2013	roach	110	-23.12	15.05	3.33	0.57	3.27
Most	2013	roach	235	-28.55	16.98	3.32	0.09	4.31

Most	2013	roach	220	-27.13	16.45	3.29	0.21	4.03
Most	2013	roach	155	-23.18	16.51	3.32	0.57	3.7
Most	2013	roach	220	-28.03	16.17	3.33	0.13	4.02
Most	2013	roach	130	-23.63	17.08	3.27	0.53	3.91
Most	2013	roach	145	-20.03	16.43	3.29	0.85	3.41
Most	2013	roach	250	-26.47	15.31	3.33	0.27	3.64
Most	2013	roach	130	-21.29	16.86	3.27	0.74	3.65
Most	2013	roach	120	-22.2	17.37	3.26	0.66	3.87
Most	2013	roach	125	-23.45	12.45	3.31	0.54	2.54
Most	2013	roach	220	-25.25	15.91	3.28	0.38	3.71
Most	2013	roach	230	-26.36	16.22	3.27	0.28	3.9
Most	2013	roach	205	-22.8	17.1	3.26	0.6	3.85
Most	2013	roach	140	-19.49	16.61	3.3	0.9	3.42
Most	2013	roach	120	-20	16.7	3.29	0.85	3.49
Most	2013	roach	235	-25.33	16.21	3.3	0.38	3.8
Most	2013	roach	115	-26.61	15.39	3.37	0.26	3.67
Most	2013	roach	110	-26.87	15.07	3.27	0.24	3.6
Most	2013	roach	225	-23.42	18.24	3.26	0.55	4.23
Most	2013	roach	240	-28.09	14.89	3.4	0.13	3.65
Most	2013	roach	210	-24.53	16.2	3.32	0.45	3.73
Most	2013	roach	140	-20.74	16.65	3.34	0.79	3.54
Most	2013	roach	125	-22.5	16.08	3.29	0.63	3.52
Most	2013	roach	210	-23.93	16.61	3.34	0.5	3.8
Most	2013	roach	55	-20.63	15.71	3.22	0.8	3.25
Most	2013	rudd	170	-24.98	13.9	3.25	0.41	3.09
Most	2013	rudd	180	-25.19	14.04	3.24	0.39	3.15
Most	2013	rudd	170	-25.1	14.2	3.28	0.4	3.19
Most	2013	rudd	165	-25.41	13.96	3.25	0.37	3.15
Most	2013	rudd	170	-24.53	13.83	3.25	0.45	3.03
Most	2013	rudd	170	-25.56	15.1	3.3	0.35	3.5
Most	2013	rudd	170	-25.34	13.82	3.27	0.37	3.1
Most	2013	rudd	180	-24.88	12.51	3.19	0.42	2.68
Most	2013	rudd	180	-25.16	14.17	3.23	0.39	3.19
Most	2013	rudd	155	-24.61	13.68	3.33	0.44	3
Most	2013	rudd	195	-24.75	13.79	3.26	0.43	3.04
Most	2013	rudd	225	-24.82	13.24	3.22	0.42	2.88
Most	2013	rudd	180	-25.5	15.55	3.27	0.36	3.62
Most	2013	rudd	165	-25.44	14.38	3.26	0.37	3.28
Most	2013	rudd	180	-25.16	14.44	3.25	0.39	3.27
Most	2013	rudd	170	-25.07	14.85	3.21	0.4	3.38
Most	2013	rudd	110	-23.94	15.73	3.27	0.5	3.54
Most	2013	rudd	100	-22.23	15.24	3.29	0.65	3.25
Most	2013	rudd	200	-23.82	14.58	3.24	0.51	3.19
Most	2013	rudd	115	-22.91	15.14	3.28	0.59	3.28
Most	2013	rudd	180	-26.69	14.99	3.25	0.25	3.56
Most	2013	rudd	115	-23.03	14.36	3.23	0.58	3.06
Most	2013	rudd	135	-23.39	15.32	3.27	0.55	3.37
Most	2013	rudd	125	-23.46	12.81	3.31	0.54	2.64
Most	2013	rudd	125	-23.9	13.68	3.25	0.5	2.94
Most	2013	rudd	175	-25.04	14.43	3.29	0.4	3.25

Most	2013	rudd	185	-24.78	15.74	3.25	0.43	3.62
Most	2013	rudd	170	-24.7	14.71	3.26	0.43	3.31
Most	2013	rudd	200	-24.49	13.83	3.27	0.45	3.03
Most	2013	rudd	175	-22.82	16.89	3.3	0.6	3.79
Most	2013	rudd	175	-25.18	15.24	3.27	0.39	3.51
Most	2014	perch	46	-24.24	18.88	3.34	0.47	4.49
Most	2014	perch	58	-27.21	14.69	3.34	0.21	3.52
Most	2014	perch	45	-25.52	16.06	3.34	0.36	3.78
Most	2014	perch	47	-22.46	16.19	3.42	0.63	3.55
Most	2014	perch	48	-22.96	15.64	3.29	0.59	3.43
Most	2014	perch	50	-26.67	14.31	3.33	0.26	3.36
Most	2014	perch	53	-20.84	16.27	3.37	0.78	3.43
Most	2014	perch	54	-23.15	14.4	3.37	0.57	3.08
Most	2014	perch	55	-24.67	16.89	3.29	0.43	3.95
Most	2014	perch	57	-25.21	13.9	3.28	0.39	3.11
Most	2014	perch	57	-26.99	14.5	3.34	0.23	3.44
Most	2014	perch	58	-21.71	15.94	3.31	0.7	3.41
Most	2014	perch	59	-22.58	15.82	3.32	0.62	3.45
Most	2014	perch	60	-26.94	14.26	3.37	0.23	3.37
Most	2014	perch	60	-26.87	14.71	3.29	0.24	3.5
Most	2014	perch	62	-25.48	15.16	3.27	0.36	3.51
Most	2014	perch	62	-26.96	13.97	3.34	0.23	3.28
Most	2014	perch	62	-22.37	15.61	3.34	0.64	3.37
Most	2014	perch	63	-26.43	13.9	3.31	0.28	3.22
Most	2014	perch	65	-24.99	14.5	3.31	0.41	3.27
Most	2014	perch	65	-26.6	14.04	3.35	0.26	3.27
Most	2014	perch	65	-22.63	15.79	3.33	0.62	3.45
Most	2014	perch	66	-26.83	13.87	3.32	0.24	3.24
Most	2014	perch	66	-25.89	15.14	3.34	0.33	3.54
Most	2014	perch	68	-26.22	13.91	3.32	0.3	3.2
Most	2014	perch	68	-26.71	14.18	3.33	0.25	3.33
Most	2014	perch	72	-22.47	15.63	3.27	0.63	3.39
Most	2014	perch	72	-26.96	13.89	3.31	0.23	3.26
Most	2014	perch	73	-26.36	13.92	3.33	0.28	3.22
Most	2014	perch	75	-28.1	13.79	3.31	0.13	3.33
Most	2014	perch	76	-24.12	15.3	3.3	0.48	3.43
Most	2014	perch	77	-26.93	13.41	3.24	0.23	3.12
Most	2014	perch	90	-24.96	14.93	3.27	0.41	3.39
Most	2014	perch	95	-24.5	15.92	3.27	0.45	3.65
Most	2014	perch	95	-20.39	16.6	3.28	0.82	3.49
Most	2014	perch	105	-22.99	15	3.27	0.59	3.25
Most	2014	perch	110	-27.35	16.07	3.27	0.19	3.94
Most	2014	perch	110	-28.2	14.69	3.56	0.12	3.6
Most	2014	perch	120	-22.52	16.39	3.3	0.63	3.61
Most	2014	perch	125	-23.39	16.87	3.27	0.55	3.83
Most	2014	perch	125	-25.04	15.53	3.3	0.4	3.58
Most	2014	perch	130	-22.92	16.34	3.31	0.59	3.63
Most	2014	perch	130	-23.41	17.24	3.27	0.55	3.94
Most	2014	perch	130	-26.6	15.86	3.25	0.26	3.81
Most	2014	perch	135	-21.91	17.6	3.26	0.68	3.92

Most	2014	perch	140	-24.8	16.16	3.28	0.42	3.74
Most	2014	perch	140	-24.67	16.26	3.26	0.44	3.76
Most	2014	perch	140	-25.06	16.02	3.25	0.4	3.72
Most	2014	perch	145	-24.71	16.19	3.19	0.43	3.74
Most	2014	perch	150	-25.77	15.7	3.22	0.34	3.69
Most	2014	perch	150	-24.43	16.42	3.25	0.46	3.79
Most	2014	perch	150	-28.45	15.89	3.25	0.1	3.98
Most	2014	perch	155	-25.95	15.82	3.24	0.32	3.74
Most	2014	perch	159	-25.74	15.78	3.17	0.34	3.71
Most	2014	perch	165	-24.42	16.19	3.19	0.46	3.72
Most	2014	perch	165	-23.32	17.5	3.3	0.56	4.01
Most	2014	perch	165	-26.11	15.6	3.27	0.31	3.69
Most	2014	perch	175	-26.13	16.1	3.28	0.3	3.84
Most	2014	perch	185	-23.26	16.14	3.26	0.56	3.6
Most	2014	perch	185	-23.58	16.83	3.28	0.53	3.83
Most	2014	perch	195	-27.36	16.62	3.33	0.19	4.1
Most	2014	perch	200	-26.73	14.53	3.33	0.25	3.43
Most	2014	perch	205	-24.08	16.44	3.17	0.49	3.76
Most	2014	perch	210	-22.16	17.05	3.2	0.66	3.78
Most	2014	perch	210	-24.63	17.08	3.25	0.44	4
Most	2014	perch	215	-24.55	17.32	3.24	0.45	4.06
Most	2014	perch	225	-26.88	16.42	3.32	0.24	4
Most	2014	perch	225	-24.63	16.9	3.3	0.44	3.94
Most	2014	perch	230	-24.35	15.81	3.21	0.46	3.6
Most	2014	perch	235	-26.91	16.2	3.42	0.23	3.94
Most	2014	perch	240	-24.07	17.18	3.28	0.49	3.98
Most	2014	perch	245	-23.65	16.68	3.27	0.53	3.79
Most	2014	perch	250	-23.38	17.74	3.23	0.55	4.09
Most	2014	perch	255	-25.97	17.19	3.27	0.32	4.15
Most	2014	perch	260	-24.07	16.84	3.25	0.49	3.88
Most	2014	perch	275	-23.04	18.35	3.2	0.58	4.23
Most	2014	perch	280	-25.04	18.08	3.28	0.4	4.33
Most	2014	perch	285	-23.35	17.25	3.32	0.55	3.94
Most	2014	perch	310	-23.25	18.11	3.31	0.56	4.18
Most	2014	perch	315	-23.93	17.88	3.19	0.5	4.17
Most	2014	perch	320	-23.89	17.72	3.36	0.5	4.12
Most	2014	perch	325	-24.49	18.65	3.19	0.45	4.45
Most	2014	perch	330	-24.02	17.76	3.23	0.49	4.15
Most	2014	perch	335	-22.1	17.67	3.28	0.67	3.95
Most	2014	perch	350	-23.47	17.75	3.15	0.54	4.1
Most	2014	perch	355	-24.24	18.62	3.21	0.47	4.42
Most	2014	perch	370	-24.77	18.38	3.24	0.43	4.39
Most	2014	perch	375	-22.74	17.98	3.23	0.61	4.1
Most	2014	roach	51	-21.99	15.38	3.35	0.68	3.27
Most	2014	roach	53	-21.44	15.72	3.34	0.72	3.32
Most	2014	roach	55	-20.99	15.54	3.33	0.77	3.23
Most	2014	roach	57	-21.72	15.19	3.34	0.7	3.19
Most	2014	roach	58	-22.07	15.24	3.3	0.67	3.24
Most	2014	roach	60	-23.94	14.45	3.31	0.5	3.17
Most	2014	roach	60	-22.32	16	3.34	0.65	3.48

Most	2014	roach	62	-23.4	15.19	3.35	0.55	3.34
Most	2014	roach	62	-24.21	15.41	3.33	0.48	3.47
Most	2014	roach	62	-23.37	14.86	3.29	0.55	3.24
Most	2014	roach	73	-21.81	15.11	3.32	0.69	3.18
Most	2014	roach	100	-20.67	13.94	3.37	0.79	2.73
Most	2014	roach	100	-20.03	15.54	3.4	0.85	3.15
Most	2014	roach	100	-20.62	15.85	3.32	0.8	3.29
Most	2014	roach	105	-20.96	16.88	3.3	0.77	3.62
Most	2014	roach	105	-20.53	15.47	3.44	0.81	3.17
Most	2014	roach	110	-20.33	19.09	3.34	0.82	4.22
Most	2014	roach	110	-18.71	16.76	3.36	0.97	3.39
Most	2014	roach	110	-20.2	16.07	3.29	0.84	3.32
Most	2014	roach	115	-21.75	16.59	3.35	0.7	3.61
Most	2014	roach	115	-20.28	17.02	3.42	0.83	3.61
Most	2014	roach	120	-21.31	14.67	3.28	0.74	3
Most	2014	roach	125	-20.17	15.48	3.32	0.84	3.14
Most	2014	roach	125	-23.54	15.49	3.3	0.54	3.44
Most	2014	roach	125	-23.11	14.97	3.42	0.57	3.25
Most	2014	roach	130	-22.4	15.68	3.25	0.64	3.39
Most	2014	roach	135	-22.1	15.37	3.54	0.67	3.28
Most	2014	roach	135	-23.18	16.13	3.35	0.57	3.59
Most	2014	roach	140	-22.03	15.65	3.31	0.67	3.35
Most	2014	roach	145	-20.59	17.36	3.42	0.8	3.73
Most	2014	roach	155	-21.85	16.68	3.42	0.69	3.64
Most	2014	roach	160	-21.22	17.45	3.2	0.74	3.81
Most	2014	roach	160	-20.62	17.4	3.3	0.8	3.75
Most	2014	roach	165	-22.96	17.75	3.37	0.59	4.05
Most	2014	roach	170	-24.08	17.06	3.28	0.49	3.94
Most	2014	roach	170	-22.17	18.23	3.37	0.66	4.13
Most	2014	roach	175	-23.35	17.32	3.31	0.55	3.96
Most	2014	roach	175	-20.74	17.87	3.35	0.79	3.9
Most	2014	roach	175	-22.83	17.31	3.31	0.6	3.91
Most	2014	roach	180	-23.87	15.36	3.29	0.51	3.43
Most	2014	roach	180	-26.07	16.54	3.29	0.31	3.96
Most	2014	roach	185	-22.57	16.65	3.29	0.62	3.69
Most	2014	roach	185	-23.65	15.34	3.31	0.53	3.4
Most	2014	roach	185	-26.09	16.73	3.33	0.31	4.02
Most	2014	roach	185	-23.7	15.34	3.36	0.52	3.41
Most	2014	roach	190	-23.14	16.54	3.35	0.57	3.71
Most	2014	roach	190	-24.55	15.05	3.32	0.45	3.39
Most	2014	roach	195	-24.1	17.8	3.27	0.49	4.16
Most	2014	roach	205	-22.77	16.23	3.34	0.61	3.59
Most	2014	roach	205	-20.37	17.34	3.37	0.82	3.71
Most	2014	roach	215	-25.57	17.14	3.28	0.35	4.1
Most	2014	roach	225	-27.39	16.12	3.53	0.19	3.95
Most	2014	roach	230	-24.9	15.56	3.33	0.41	3.57
Most	2014	roach	230	-24.95	17.79	3.3	0.41	4.23
Most	2014	roach	230	-24	15.77	3.29	0.5	3.56
Most	2014	roach	230	-26.65	16.19	3.27	0.26	3.91
Most	2014	roach	230	-22.39	16.81	3.29	0.64	3.73

Most	2014	roach	230	-28.15	16.57	3.32	0.12	4.15
Most	2014	roach	240	-27.03	16.35	3.33	0.22	3.99
Most	2014	roach	240	-22.46	16.48	3.48	0.63	3.63
Most	2014	roach	240	-28.61	16.08	3.3	0.08	4.05
Most	2014	roach	240	-27.05	14.91	3.51	0.22	3.57
Most	2014	roach	245	-28.22	16.48	3.35	0.12	4.13
Most	2014	roach	250	-28.36	16.46	3.26	0.1	4.14
Most	2014	roach	260	-26.75	15.47	3.32	0.25	3.71
Most	2014	roach	270	-28.45	16.27	3.42	0.1	4.09
Most	2014	rudd	51	-22.71	14.39	3.41	0.61	3.04
Most	2014	rudd	83	-22.75	15.23	3.3	0.61	3.29
Most	2014	rudd	85	-22.92	14.32	3.32	0.59	3.04
Most	2014	rudd	89	-22.42	14.09	3.34	0.64	2.93
Most	2014	rudd	90	-22.19	15.78	3.33	0.66	3.41
Most	2014	rudd	95	-22.59	15.11	3.31	0.62	3.24
Most	2014	rudd	95	-23.35	14.62	3.29	0.55	3.16
Most	2014	rudd	100	-22.32	15.12	3.22	0.65	3.22
Most	2014	rudd	120	-22.84	14.22	3.29	0.6	3
Most	2014	rudd	120	-27.52	16.39	3.53	0.18	4.05
Most	2014	rudd	125	-23.43	15.13	3.28	0.55	3.32
Most	2014	rudd	130	-23.35	13.96	3.21	0.55	2.97
Most	2014	rudd	130	-23.02	14.68	3.29	0.58	3.15
Most	2014	rudd	135	-24.31	14.6	3.19	0.47	3.24
Most	2014	rudd	140	-22.58	15.54	3.21	0.62	3.37
Most	2014	rudd	140	-22.44	14.83	3.28	0.63	3.15
Most	2014	rudd	140	-21.86	14.28	3.29	0.69	2.94
Most	2014	rudd	145	-23.31	14.45	3.38	0.56	3.11
Most	2014	rudd	150	-24.01	14.71	3.21	0.49	3.25
Most	2014	rudd	155	-24.06	14.53	3.27	0.49	3.2
Most	2014	rudd	170	-23.02	13.07	3.24	0.58	2.68
Most	2014	rudd	180	-22.45	12.18	3.24	0.63	2.37
Most	2014	rudd	195	-23.68	12.3	3.33	0.52	2.51
Most	2014	rudd	195	-24.21	14.33	3.25	0.48	3.15
Most	2014	rudd	200	-24.9	13.31	3.32	0.41	2.91
Most	2014	rudd	205	-25.51	13.5	3.27	0.36	3.02
Most	2014	rudd	235	-27.38	15.78	3.78	0.19	3.85
